# Hormone crosstalk in plants

**DOI:** 10.1093/jxb/erv339

**Published:** 2015-07-24

**Authors:** Angus Murphy

**Affiliations:** University of Maryland, USA

For years, many plant science researchers who study the genetic programming that determines structural organization and reproductive processes self-identified as developmental biologists, while those studying plant responses to the environment identified as plant physiologists. Plant hormones such as auxins, cytokinins, and gibberellic acids that function as developmental messengers were viewed as functionally distinct from ‘stress’ and ‘defence’ hormones such as ethylene, abscisic acid, jasmonic acid, and salicylic acid. This division of hormone function was convenient, but was also known to be thoroughly artificial. Over the past two decades, new hormones have been identified, tissue and organ-specific hormone functions have been determined, methods have been developed to measure and visualize hormones *in situ*, receptor mechanisms have been conclusively identified or discounted, hormone transport processes have been largely elucidated, and the cellular processes downstream of hormone signalling have been painstakingly dissected.

Over time, simple models of hormone antagonism (gibberellic acid vs. abscisic acid, auxin vs. cytokinin) have been displaced by the concept of complex hormonal crosstalk. The classification of hormones into developmental or environmental response categories has been replaced by mapping of hormonal signalling into transcriptional and post-transcriptional response networks. This transition has accompanied a profound shift in the way that plant physiology and development are taught in the classroom, with undergraduate and graduate courses increasingly combining physiology and development as inseparable components of growth. Book chapters and reviews that were previously focused on specific hormones now address aspects of programmed and plastic development and their by genetics, epigenetics, protein modification, second messengers, and hormones. This special issue on Hormone Crosstalk is intended to provide an update of the topic that will be of value to both experts in the field and the general community of plant scientists.

The reviews and original research articles presented in this special issue were invited in consultation with the organizers of the 2014 International Symposium on Auxin and Cytokinins in Development (ACPD) held in Prague, Czech Republic. For the past 15 years, the ACPD conference has been organized by the Institute of Experimental Botany/Academy of Sciences of the Czech Republic to provide a primary forum for scientific discussions of recent progress in the understanding of hormonal control of plant development. As is always the case with the Journal, all of the articles accepted for this special issue were rigorously reviewed by external experts to assure that they meet journal standards.

The issue begins with six review articles (Zwack and Rashotte, 2015; Schäfer *et al.*, 2015; Naseem *et al.*, 2015; Hrtyan *et al.*, 2015; Zdarska *et al.*, 2015; and Koltai, 2015) describing various aspects of hormonal crosstalk observed in developmental and environmental plant responses. Next are three reviews (Zhu and Geisler, 2015; Hill, 2015; and Turchi *et al.*, 2015) that provide updates on mechanisms that function in hormonal signalling. Three more reviews (Doyle *et al.*, 2015; Fraas and Lüthen, 2015; and Chen *et al.*, 2015) describe improved experimental systems used to characterize hormone function. Reviews by Cortleven and Schmülling (2015); Didi *et al.* (2015); and Robert *et al.* (2015) provide updates on hormonal interactions that regulate chloroplast development, cell wall formation and organogenesis, respectively.

Six invited original research articles round out this special issue. Grones *et al.* (2015) analyse auxin binding pocket function in gain-of-function *Auxin Binding Protein 1* lines in an effort to reassess the function of this controversial protein. New approaches to modelling of polarized auxin fluxes are described by Cieslak *et al.* (2015). Löfke et al. (2015) characterize the relative contributions of PIN2 auxin efflux carrier activity in atrichoblasts and root hair cell files. The role of chaperone proteins in maintaining auxin flows is expanded by a study from D’Alessandro *et al.* (2015) in which the p23 chaperone protein is shown to regulate auxin-dependent root development and meristem function. Song *et al.* (2015) characterize interactions between cytokinin biosynthetic genes and nutrient mobilization proteins in *Brassica napus* development. Finally, Stes *et al.* (2015) describe a previously undescribed function of strigolactones in the leafy gall syndrome of *Arabidopsis*.

It is our hope that this set of articles will provide the reader with an enhanced understanding of hormonal crosstalk and stimulate new avenues of research in this emergent area of inquiry.
